# Multicomponent Peptide-Based Hydrogels Containing Chemical Functional Groups as Innovative Platforms for Biotechnological Applications

**DOI:** 10.3390/gels9110903

**Published:** 2023-11-15

**Authors:** Sabrina Giordano, Enrico Gallo, Carlo Diaferia, Elisabetta Rosa, Barbara Carrese, Nicola Borbone, Pasqualina Liana Scognamiglio, Monica Franzese, Giorgia Oliviero, Antonella Accardo

**Affiliations:** 1 Department of Pharmacy, University of Naples “Federico II”, Via D. Montesano 49, 80131 Naples, Italy; sabrina.giordano@unina.it (S.G.); carlo.diaferia@unina.it (C.D.); nicola.borbone@unina.it (N.B.); 2 IRCCS SYNLAB SDN, Via Gianturco 113, 80143 Naples, Italy; enrico.gallo@synlab.it (E.G.); barbara.carrese@synlab.it (B.C.); monica.franzese@synlab.it (M.F.); 3 Department of Sciences, University of Basilicata, Via dell’Ateneo Lucano 10, 85100 Potenza, Italy; 4 Department of Molecular Medicine and Medical Biotechnologies, University of Naples “Federico II”, Via S. Pansini 5, 80131 Naples, Italy; giorgia.oliviero@unina.it

**Keywords:** Fmoc-FF, peptide materials, hydrogels, peptide self-assembling, soft materials

## Abstract

Multicomponent hydrogels (HGs) based on ultrashort aromatic peptides have been exploited as biocompatible matrices for tissue engineering applications, the delivery of therapeutic and diagnostic agents, and the development of biosensors. Due to its capability to gel under physiological conditions of pH and ionic strength, the low molecular-weight Fmoc-FF (N^α^-fluorenylmethoxycarbonyl-diphenylalanine) homodimer is one of the most studied hydrogelators. The introduction into the Fmoc-FF hydrogel of additional molecules like protein, organic compounds, or other peptide sequences often allows the generation of novel hydrogels with improved mechanical and functional properties. In this perspective, here we studied a library of novel multicomponent Fmoc-FF based hydrogels doped with different amounts of the tripeptide Fmoc-FF*X* (in which *X*= Cys, Ser, or Thr). The insertion of these tripeptides allows to obtain hydrogels functionalized with thiol or alcohol groups that can be used for their chemical post-derivatization with bioactive molecules of interest like diagnostic or biosensing agents. These novel multicomponent hydrogels share a similar peptide organization in their supramolecular matrix. The hydrogels’ biocompatibility, and their propensity to support adhesion, proliferation, and even cell differentiation, assessed in vitro on fibroblast cell lines, allows us to conclude that the hybrid hydrogels are not toxic and can potentially act as a scaffold and support for cell culture growth.

## 1. Introduction

**Figure 1:**
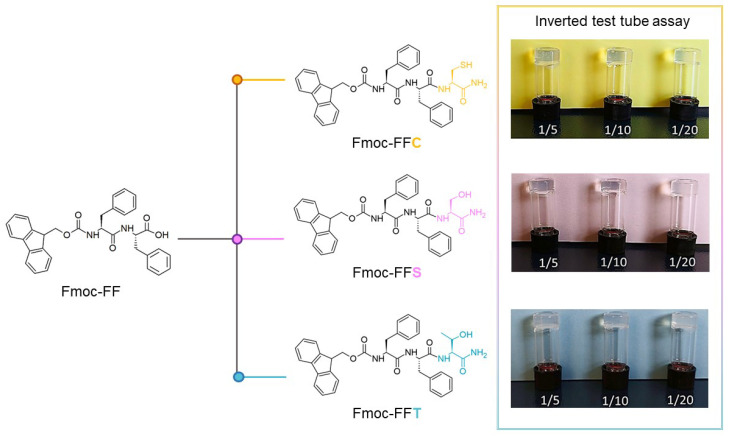
Chemical structure of peptide sequences Fmoc-FFC, Fmoc-FFS, and Fmoc-FFT, and theinverted test tube of mixed hydrogels formulated by combining these peptides with Fmoc-FF at different molar ratios.

In recent years, many different materials such as nanofibers [1,2], nanotubes [3,4], nanospheres [5,6],and hydrogels (HGs) [7,8] have attracted increasing interest as suitable and innovative platforms for biomedical and pharmaceutical applications [9,10,11], including tissue engineering [12,13] and the delivery of drugs [14,15] or diagnostic agents [16,17]. Hydrogels are three-dimensional macroscopic networks able to retain large amounts of water or other biological fluids [18]. Despite the massive amount of liquid included, hydrogels are able to display solid-like viscoelastic properties, and the presence of the supramolecular network allows the entire system to appear self-supporting [19,20]. These materials can be obtained through self-assembling of polymers or smaller molecules able to self-organize themselves upon a trigger stimulus [21]. Due to their capability to establish non-covalent interactions and to assume secondary structure elements, peptides have been also identified as potential building blocks for generating hydrogels [22]. Among the different self-assembling classes of peptides (e.g., amphipathic, surfactant-like, lipidated, bolamphiphiles, cyclic, and aromatic), the low molecular-weight Fmoc-FF (N*^α^*-fluorenylmethoxycarbonyl-diphenylalanine) homodimer is one of the most studied [23,24]. The interest around this hydrogelator arises thanks to its ability to gel under physiological conditions of pH and ionic strength [25]. Other additional advantages provided by this peptide are its very simple nature, the high synthetic accessibility, and the low cost. The gel properties of Fmoc-FF have been exploited for the peptide alone or in combination with other components (like natural or synthetic polymers, peptides, polysaccharides, or organic molecules), since it has been demonstrated that their inclusion into the matrix can improve and tune the physiochemical features of the final material [26,27,28,29]. From this perspective, here the gelation properties exhibited by Fmoc-FF in combination with three of its tripeptide analogs at different weight/weight ratios (1/5, 1/10, and 1/20 *w*/*w*) were studied. In these tripeptides, Fmoc-FFX, the C-terminus of the Fmoc-FF was derivatized with a cysteine (X= Cys, C), serine (X = Ser, S) or threonine (X = Thr, T) residue (Figure 1). The first two amino acids, Cys and Ser, differ from each other only by the heteroatom (sulfur and oxygen, respectively) in their side chain, whereas Thr has an additional methyl group on the C*_β_* respect to the Ser. The three residues were rationally chosen, to install in the final matrix a functional group (like thiol or alcohol one) that can be used for the chemical post-derivatization of the matrix with bioactive molecules of interest. The development of novel biomaterials doped with bioactive agents, including therapeutics [30] and diagnostics [31], is closely linked to different biomedical applications that span tissue engineering [32,33], drug delivery [34], biosensors [35], diagnostics [36], and theranostics [37]. These kinds of nanomaterials are expected to provide a novel platform for personalized medicine, which may change the future shape of the pharmaceutical industry [38]. The hybrid systems here designed were fully characterized from the structural and mechanical point of view. The supramolecular behavior of the tripeptides alone, or in combination with Fmoc-FF, was investigated using a set of techniques (rheology, FT-IR, CD, SEM, fluorescence, and optical microscopies). Results demonstrated that the novel multicomponent hydrogels share a similar peptide organization in their supramolecular matrix. The biocompatibility and propensity to support adhesion, proliferation, and even differentiation of cells of the supramolecular structures were assessed in vitro on fibroblast cell lines.

## 2. Results and Discussion

### 2.1. Design and Synthesis of Peptide Building Blocks

Fmoc-FF represents the paradigmatical example of an all-aromatic peptide-based hydrogelator. The synthetic accessibility of peptides allows modification of the sequence, adding chemical functionalities by simply altering the primary sequence. The structural importance of the mutual proximity of Fmoc groups and Phe residues in the Fmoc-FF gelation suggested the designed Fmoc-FFX short sequence, in which the additional residue (X) is positioned at the end of the Fmoc-FF motif. This design should assure a high chemical homology, coupled with a slight perturbance of the non-covalent interaction pathways responsible for both aggregation and gelation. Among the 20 natural amino acids, Cys, Ser, and Thr were selected to allow the inclusion of the corresponding thiol (Cys), or primary (Ser) or secondary (Thr), alcohol functional groups. These functional groups may be suitable for post-aggregative functionalization, permitting the development of responsive systems useful for diagnostic or analyte detection [39]. The synthesis and purification of the three novel tripeptides (Fmoc-FFC, Fmoc-FFS, and Fmoc-FFT, reported in Figure 1) were achieved by standard solid-phase peptide synthesis and RP-HPLC chromatography, respectively. Their identity was then assessed by analytical HPLC characterization, ESI mass spectrometry, and ^1^H-NMR spectroscopy (Table 1 and Figures S1–S6). 

### 2.2. Hydrogel Formulation

These peptides were used to dope Fmoc-FF hydrogels. However, their ability to self-gel alone was also checked. The self-gelification of each peptide was studied under different conditions of concentration, using the solvent-switch method (DMSO/H_2_O). This method allows gel formation by adding the desired amount of water to a peptide solution previously dissolved in DMSO at a concentration of 100 mg·mL^−1^. Under the studied experimental conditions, no formation of self-supporting hydrogels was observed. Consequently, the formulation of mixed matrices was evaluated, combining Fmoc-FFX sequences with Fmoc-FF in several weight/weight percentages (1/5, 1/10, and 1/20 *w/w* for Fmoc-FFX/Fmoc-FF, where X = C, S, T). The amount of the tripeptide was intentionally decreased in the mixed matrices, in progressive way, to fabricate hydrogels with a different functionalization degree. Analogously, hybrid hydrogels were also prepared by the solvent-switch method by keeping constant the *ϕ*_DMSO/H2O_ (10/90). As expected for Fmoc-FF, a transition of the metastable solution from opaque to limpid occurs after the addition of water. Due to the decrease in the refractive index related to the gelation process, a quantitative determination of the gelation kinetics can be performed by measuring the absorbance spectrum of the sample at 600 nm (long-range channel) over time at selected time points. All the samples show a decrease in the optical density (OD) during the opaque-to-limpid transition [40], and the gelation times (Gts) were estimated in the flex of the profiles (Figure S7). From the inspection of the times in Table 2, it can be observed that the gelation kinetics are affected by the amount of the tripeptide respect to Fmoc-FF, with an increase in the gelation time for increasing the amount of Fmoc-FFX tripeptides in the mix. However, it is kepta similar trend for each *w/w* ratio, with a Gt_(Fmoc-FFT)_> Gt_(Fmoc-FFS)_> Gt_(Fmoc-FFC)_, thus suggesting that the different steric hindrance and heteroatom can affect the gel formation. 

The shelf stability and degradability of the mixed hydrogels was assessed through the inverted test tube (Figure 1) and the swelling test (Table 2 and Figure S8). The swelling percentage found for the hybrid gels ranged between 33% and 41%. As expected for mixed hydrogels, these values are higher than the swelling percentage determined for the pure Fmoc-FF hydrogel (29%) [28].

### 2.3. Secondary Structural Characterization

**Figure 2:**
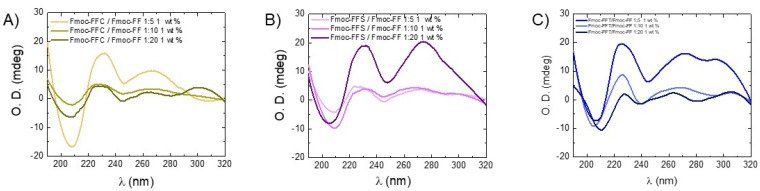
CD spectra of mixed hydrogels Fmoc-FFX/Fmoc-FF at 1/5, 1/10, and 1/20 *w*/*w* ratios. (**A**) X = Cys; (**B**) X = Ser; (**C**) X = Thr.

To rationalize the capability of the tripeptides to perturb the Fmoc-FF organization into the hydrogel, mixed HGs were characterized by Circular Dichroism and Fourier Transform Infrared spectroscopies. The structural evidence arising from CD and FT-IR characterization were further confirmed by ThT and CR assays. Previous structural and molecular dynamic simulation studies reported in the literature demonstrated that, in the Fmoc-FF hydrogels, the peptide segments self-assemble into *β*-sheet with an antiparallel orientation of the *β*-strands [41]. It was also reported that the further hierarchical organization of the *β*-sheet in Fmoc-FF nanostructures causes the formation of superhelical architectures [42]. As can be deduced from an inspection of Figure 2, no significant differences are revealed among tested samples and by comparing them with the CD profile of the hydrogel composed only by Fmoc-FF. Indeed, for all of them, the presence of a positive band around 195 nm and a negative one centered between 205 and 210 nm is detected and can be ascribed to π→π* *α*-helix transitions [25,43]. The presence of a Cotton effect around 230 nm, attributed to n-π* transitions, is indicative of a super-helical arrangement of the phenylalanine residues. This arrangement causes, in the hydrogels, a helical orientation of the fluorenyl moieties, as assessed by the broad band around 270–280 nm [44]. These results thus suggest that the supramolecular organization of the matrices is driven by Fmoc-FF moiety. However, the signal intensity decreases with the increase in Fmoc-FF concentration. This slight difference in the CD signal can be probably attributed to the increasing opacity of the hydrogels as a function of the Fmoc-FF amount. 

**Figure 3:**
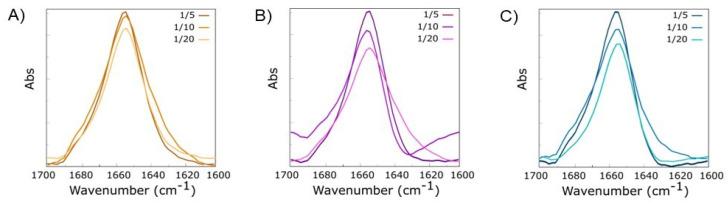
FT-IR spectra in the Amide I region of mixed hydrogels Fmoc-FFX/Fmoc-FF at 1/5, 1/10, and 1/20 *w/w* ratios. (**A**) X = Cys; (**B**) X = Ser; (**C**) X = Thr.

The super-helix arrangements of mixed hydrogels were also confirmed by FT-IR spectroscopy analysis. FT-IR is a conservative technique sensitive to the secondary structure of proteins and peptides, widely used to investigate both misfolding and aggregate formation [45,46]. Specifically, the C=O stretching bands are collected in the amide I region (1700–1600 cm^−1^). These signals are sensible to specific secondary structure arrangements, related to both hydrogen bonding networks and tridimensional molecular organization [47]. Consequently, the band wavenumbers in Amide I directly correlate to the protein’s secondary structure elements. Amide Iregion FT-IR spectra of mixed HGs in water are collected in Figure 3 and reported as an absorbance deconvolution. An intense band (centered at 1656 cm^−1^) governs all the spectra independently from HGs’ chemical composition and the constituents’ relative ratio. This band is generally attributed to helix conformation of the protein or a super-helix supramolecular structure [47]. 

**Figure 4:**
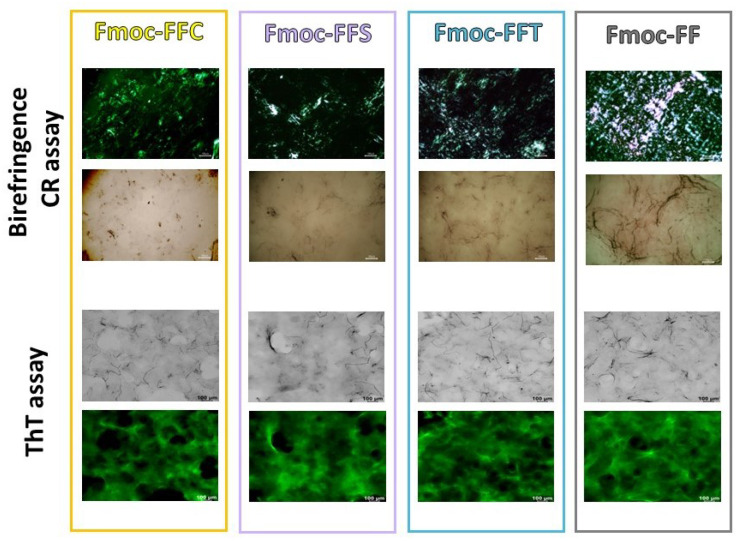
Birefringence CR assay and ThT assays. Polarized optical microscopy images of air-dried samples stained with Congo Red solution between crossed polarizers and under bright-field illumination (first and second rows). Confocal images of xerogels, stained with 50 μmol/L ThT solution, under bright field and in the green spectral region (GFP), *λ*_exc_ = 488 nm (third and fourth rows).

For all the samples, the absorbance signal’s intensity is inversely proportional to the Fmoc-FF relative amount in mixed gels. This evidence may be related to the different transmittance and opacity of the samples, as supported by the macroscopic appearance of gels (see Figure 1, inverted test tube). Further information on the secondary structure of peptides in our mixed gels was obtained by performing the birefringence of CR and ThT assay on the xerogels, prepared by drying gels on the slide glass. Due to their capability to interact with amyloid-like fibers, CR and ThT dyes are commonly employed to detect them, both in solution and in the solid state [48,49]. In particular, CR, upon interaction with fibers rich in *β*-sheet structures, can exhibit a typical apple-green birefringence under cross-polarized light. On the other hand, ThT, which does not emit fluorescence in water, can emit in the green spectral region (~480 nm) in the presence of *β*-sheet structures. In Figure 4, it can be observed that xerogels stained with CR and ThT are positive to both the assays, thus confirming the secondary structure of the peptides into the mixed matrices. 

### 2.4. SEM Characterization

**Figure 5:**
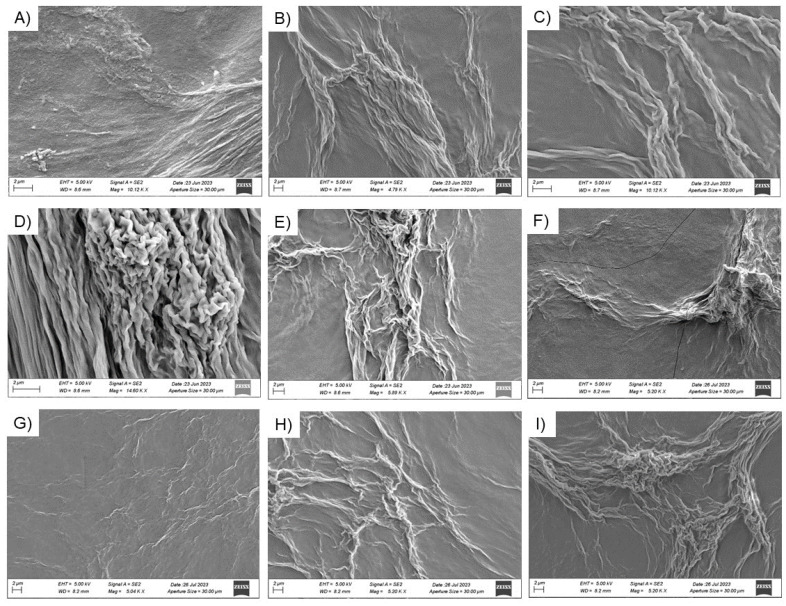
Selected microphotos of mixed xerogels: Fmoc-FFC/Fmoc-FF at (**A**) 1/5, (**B**) 1/10, and (**C**) 1/20; Fmoc-FFS/Fmoc-FF at (**D**) 1/5, (**E**) 1/10, and (**F**) 1/20 *w/w* ratios; Fmoc-FFT/Fmoc-FF at (**G**) 1/5, (**H**) 1/10, and (**I**) 1/20 *w/w* ratios. Scale bar 2 µm.

The characterization of supramolecular elements forming the hydrogels matrices, in terms of topography and morphology, was assessed using SEM. Representative SEM microphotos, collected in Figure 5, show the presence of elongated unbranched fibers for all the samples. The supramolecular fibrillar elements are also characterized by a right-handed twisting. This mutual entanglement of fibers is typically observed for peptide-based hydrogels [50]. 

### 2.5. Rheological Characterization

The mechanical properties of mixed hydrogels were investigated by rotational rheological analysis.

**Figure 6:**
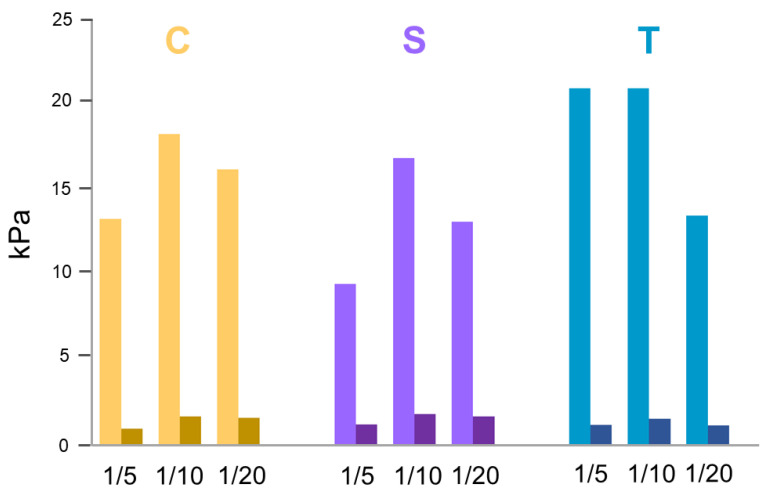
Histogram of rheological analysis for mixed hydrogels. G′ (storage modulus) is reported in light color, G″ (loss modulus) in its dark analog.

Before starting with measurements, the experimental parameters were optimized via dynamic oscillation strain sweep (at a frequency of 1.0 Hz) and dynamic frequency sweep (at 0.1% strain) (data not shown). The linear viscoelastic region (LVR) was identified in the 0.01% <*ω* < 7.0%. The time sweeps oscillatory measurements (20 min, 1.0 Hz and 0.1 % strain, Figure 6 and Figure S9) were carried out on the preformed sample for each mixed gel. From the inspection of the Figure, it can be observed that the G′ (storage modulus) value is always higher than the G″(loss modulus) one, thus analytically confirming that all the studied samples are in the gel state (see Table 2). From the comparison of values in Table 2, it can be concluded that, for all the samples, the insertion of the tripeptide into the Fmoc-FF allows a rigidification of the gel. This result is not surprising, if compared to other peptide-based mixed hydrogels described in the literature [28,51]. Indeed, it has been demonstrated that, in multicomponent matrices, peptides can establish among them additional interactions that cause an increase in the mechanical response. Moreover, the tan*δ* (G′/G″), ranging between 7.8 and 17.8, also suggests a prominent viscoelastic nature of the gel with the creation of a very strong matrix. 

### 2.6. Cytotoxicity and Cell Adhesion Assays

**Figure 7:**
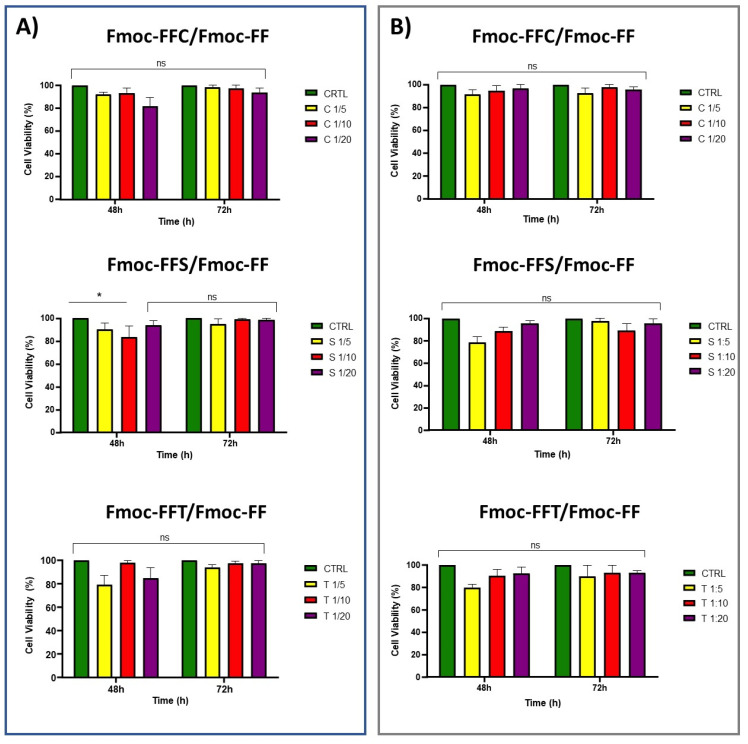
MTS assay of (**A**) HaCat and 3T3-L1 (**B**) cell lines treated for 48 and 72 h with hydrogels-conditioned medium. * *p* < 0.05 versus control cells.

**Figure 8:**
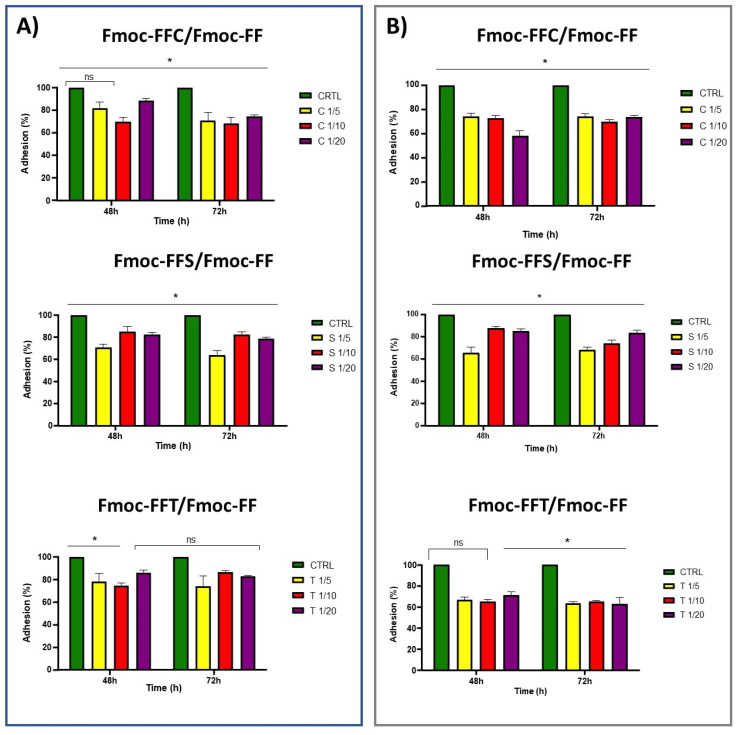
Adhesion test of (**A**) HaCat and 3T3-L1 (**B**) cell lines for 48 and 72 h on indicated mixed hydrogels. * *p* < 0.05, versus control cells.

The in vitro cytotoxicity of mixed hydrogels was evaluated on two different fibroblast cell lines, derived from human (HaCat) and from mouse (preadipocyte 3T3-L1), using the MTS assay. Cell viability was tested in the presence of the conditioned medium incubated with the hydrogel. To allow this, before any cell experiments, hydrogels were put in contact with DMEM for 16 h. After this time, cells were incubated with the conditioned medium, and, as it is shown in Figure S8, a slight toxicity on the HaCat cell line was observed after 24 h. This toxicity was previously detected on MDA-MB-231 cell lines similarly treated with peptide hydrogels and was demonstrated to be related to a cell cycle arrest at the S phase [52]. However, after 48 and even 72 h, a negligible or no toxicity, in respect to the control, can be observed for all the tested samples on both cell lines (Figure 7). To test the ability of hydrogels to support the in vitro growth of eukaryotic cells, HaCat and 3T3-L1 cell lines were cultured on our novel synthetic hydrogels. Cells were plated on pre-casted hydrogels, and the adhesion efficiency was measured up to 72 h after seeding, and then compared to cells plated in the absence of hydrogels. As shown in Figure 8, HaCat cells showed a good adhesion level up to 72 h of about 71%, 68%, and 75% when incubated with Fmoc-FFC/Fmoc-FF mixed hydrogels at 1/5, 1/10, and 1/20 *w/w*, respectively, while 3T3-L1 cells showed a 74%, 70%, and 72% of adhesion on Fmoc-FFC/Fmoc-FF mixed hydrogels at 1/5, 1/10, and 1/20 *w/w*. On the contrary, HaCat cells plated on Fmoc-FFS/Fmoc-FF mixed hydrogels at 1/5, 1/10, and 1/20 *w/w* adhere up to 72 h to hydrogels showing results of 64%, 82%, and 77%, respectively, while 3T3-L1 showed 68%, 74%, and 83% of adhesion to the same hydrogels. 

At the end, HaCat cells adhere to Fmoc-FFT/Fmoc-FF mixed hydrogels at 1/5, 1/10, and 1/20 *w/w* up to 72 h for results of 64%, 82%, and 77% respectively; on the contrary, 3T3-L1 cells showed a 63%, 65%, and 63% of adhesion in the same conditions. In conclusion, the results showed that adhered cells on any of these tested hydrogels remained viable up to 72 h.

## 3. Conclusions

Peptides and peptidomimetics have been identified as suitable building blocks for the development of supramolecular platforms such as nanotubes, fibers, and hydrogels. Their *ad-hoc* modification with chemical or biological entities like peptide nucleic acids, fluorophores, antibodies, and therapeutic and diagnostic agents could allow the obtaining of novel materials with enhanced properties for biomedical applications. Particularly appealing is the possibility of achieving the derivatization of the hydrogel via a post-gelation modification. In this perspective, it was studied the suitability of Fmoc-FF based hydrogel to generate mixed hydrogels, in combination with tripeptides sharing the same aromatic core of Fmoc-FF and bearing an additional residue of Cys, Ser, or Thr at their C-terminus. The structural, mechanical, and morphological properties of these mixed matrices were assessed, evaluating the effects caused by their different side chains. Materials with the desired degree of reactive groups on their surface can be easily obtained by doping mixed hydrogels with different amounts of the reactive function. 

## 4. Materials and Methods

### 4.1. Experiment

Protected N^α^-Fmoc-amino acids, coupling reagents and Rink amide MBHA (4-methylbenzhydrylamine) resin were purchased from Calbiochem-Novabiochem (Läufelfingen, Switzerland). Fmoc-FF peptide was bought fromBachem (Bubendorf, Switzerland). All other chemical products are commercially available from Merck (Milan, Italy), Fluka (Bucks, Switzerland), or LabScan (Stillorgan, Dublin, Ireland) and, unless stated otherwise, were used as delivered by the companies. Samples and peptide-based hydrogels were prepared by weight using dimethylsulfoxide (DMSO) and double-distilled water. Purification of crude peptides was carried out via preparative RP-HPLC chromatography on a Phenomenex (Torrance, CA, USA) C18 column using an LC8 Shimadzu HPLC system (Shimadzu Corporation, Kyoto, Japan) equipped with an UV lambda-Max Model 481 detector. Elution solvents were H_2_O/0.1% trifluoroacetic acid (TFA) (A) and CH_3_CN/0.1% TFA (B) from 20% to 90% over 30 min at a flow rate of 20 mL·min^−1^. The purity of the products was assessed via analytical reversed-phase high-performance liquid chromatography (RP-HPLC) analysis performed using Finnigan Surveyor MSQ single quadrupole—positive electrospray ionization (Finnigan/Thermo Electron Corporation San Jose, CA, USA), with a C18-Phenomenex column eluting with H_2_O/0.1% TFA (A) and CH_3_CN/0.1% TFA (B) from 20% to 80% over 20 min at a flow rate of 1 mL·min^−1^. ESI sources parameters: sheath gas flow rate (arb.) = 8; I spray voltage = 3.50 kV; spray current = 1.20 μA; capillary temperature = 275 °C; capillary voltage = 31 V; tube lens = 100 V. The identity of peptides was assessed by mass spectrometry using a LTQ XL Linear Ion Trap Mass Spectrometer, ESI source.

### 4.2. Solid Phase Peptide Synthesis

Peptides were synthesized according to standard solid-phase peptide synthesis (SPPS) procedures using the Fmoc/*t*Bu strategy [53]. Briefly, the solid support, Rink amide MBHA resin (substitution 0.70 mmol g^−1^) was swelled in *N,N*-dimethylformamide (DMF) for 45 min. Then, the Fmoc protecting group was removed, using twice (each treatment for 8 min) a solution of 20% *v*/*v* piperidine in DMF. Successively, each amino acid was in sequence coupled on the resin. This step was performed by treating the resin for 45 min with the corresponding Fmoc-protected amino acid (2 equivs.) dissolved in DMF. The reaction was activated using 2 equivalents of 1-hydroxybenzotriazole (HOBt) and benzotriazol-1-yl-oxytris-pyrrolidino-phosphonium (PyBOP) and 4 equivalents of di-isopropylethylamine (DIPEA). At the end of the synthesis, each peptide was cleaved from the resin by leaving it under stirring for 2 h into a solution of TFA/triisopropylsilane/1,2-ethanedithiol/ H_2_O (92.5/2.5/2.5/2.5 *v/v/v/v*). The peptides underwent a precipitation in cold ether and three cycles of lyophilization. The purity of crude peptides was found to be in the 80–90% range. 

### 4.3. Peptide Characterization via HNMR Spectroscopy

#### 4.3.1. Fmoc-FFC

H-NMR (700 MHz, DMSO) (chemical shifts in *δ*, DMSO-d_6_ as internal standard 2.55). δ 8.26 (d, *J* = 7.8 Hz, 1H), 8.12 (d, *J* = 8.0 Hz, 1H), 7.88 (dd, *J* = 7.6, 2.4 Hz, 2H), 7.63 (d, *J* = 7.5 Hz, 1H), 7.61 (dd, *J* = 8.1, 4.7 Hz, 2H), 7.41 (q, *J* = 6.9 Hz, 2H), 7.37–7.30 (m, 1H), 7.29–7.21 (m, 11H), 7.19–7.14 (m, 2H), 4.62–4.56 (m, 1H), 4.34 (td, *J* = 7.6, 5.0 Hz, 1H), 4.23 (ddd, *J* = 10.8, 8.7, 3.9 Hz, 1H), 4.16 (dd, *J* = 7.3, 4.6 Hz, 1H), 4.15–4.07 (m, 2H), 3.08 (dd, *J* = 14.0, 5.0 Hz, 1H), 2.93 (dd, *J* = 13.8, 3.8 Hz, 1H), 2.88 (dd, *J* = 14.0, 9.0 Hz, 1H), 2.81 (ddd, *J* = 13.8, 8.9, 5.0 Hz, 1H), 2.73 (ddd, *J* = 13.7, 10.4, 6.8 Hz, 2H), 2.24 (t, *J* = 8.3 Hz, 1H).

#### 4.3.2. Fmoc-FFS

H-NMR (700 MHz, DMSO) (chemical shifts in *δ*, DMSO-d_6_ as internal standard 2.55). δ 8.19 (d, *J* = 8.0 Hz, 1H), 8.01 (d, *J* = 7.9 Hz, 1H), 7.88 (dd, *J* = 7.7, 2.4 Hz, 3H), 7.63 (d, *J* = 7.5 Hz, 1H), 7.60 (t, *J* = 8.9 Hz, 2H), 7.41 (q, *J* = 7.1 Hz, 3H), 7.32 (t, *J* = 7.4 Hz, 2H), 7.30–7.20 (m, 11H), 7.19–7.08 (m, 4H), 4.61 (td, *J* = 8.4, 4.5 Hz, 1H), 4.27–4.19 (m, 2H), 4.16 (td, *J* = 8.6, 6.0 Hz, 1H), 4.14–4.06 (m, 2H), 3.95 (s, 1H), 3.62 (dd, *J* = 10.7, 5.4 Hz, 1H), 3.56 (dd, *J* = 10.8, 5.1 Hz, 1H), 3.08 (dd, *J* = 14.0, 4.8 Hz, 1H), 2.93 (dd, *J* = 13.8, 3.9 Hz, 1H), 2.85 (dd, *J* = 14.1, 9.1 Hz, 1H), 2.71 (dd, *J* = 13.8, 10.9 Hz, 1H).

#### 4.3.3. Fmoc-FFT

H-NMR (700 MHz, DMSO) (chemical shifts in *δ*, DMSO-d_6_ as internal standard 2.55). δ 8.29 (d, *J* = 8.0 Hz, 1H), 7.88 (dd, *J* = 7.6, 2.7 Hz, 3H), 7.80 (d, *J* = 8.7 Hz, 1H), 7.63 (d, *J* = 7.5 Hz, 1H), 7.60 (dd, *J* = 10.4, 8.2 Hz, 2H), 7.41 (q, *J* = 7.1 Hz, 3H), 7.35–7.30 (m, 1H), 7.30–7.21 (m, 11H), 7.19–7.11 (m, 4H), 7.02–6.97 (m, 1H), 4.91 (d, *J* = 4.9 Hz, 1H), 4.68–4.63 (m, 1H), 4.26–4.20 (m, 1H), 4.16 (dd, *J* = 8.6, 5.9 Hz, 1H), 4.10 (dtd, *J* = 15.6, 8.1, 2.3 Hz, 3H), 4.07–4.01 (m, 1H), 3.93 (s, 1H), 3.09 (dd, *J* = 14.1, 4.8 Hz, 1H), 2.95–2.90 (m, 1H), 2.87 (dd, *J* = 14.0, 9.4 Hz, 1H), 2.71 (dd, *J* = 13.8, 10.9 Hz, 1H), 1.02 (d, *J* = 6.3 Hz, 3H).

### 4.4. Hydrogels Formulation

Self-assembled hydrogels were formulated *via* DMSO/H_2_O solvent-switch method by using different weight/weight percentages: 0.25 wt %, 0.50 wt %, 1.0 wt %, and 2.0 wt %. Mixed hydrogels were formulated at a concentration of 1.0 wt % (10 mg·mL^−1^) *via* DMSO/H_2_O solvent-switch method using different ratios of the three different peptides and of the aromatic dipeptide Fmoc-FF. The examined ratios for mixed hydrogels were 1/5, 1/10, and 1/20 *w/w*. Stock solutions of each peptide were prepared in DMSO (100 mg·mL^−1^), then mixed, vortexed, and subsequently rehydrated with water. During the rehydration step, the mixtures were additionally stirred for 2s to promote the homogeneity of the samples. The formation of hydrogels was macroscopically assessed *via* inverted test tube.

### 4.5. Hydrogel Swelling Studies

The swelling ratios of hydrogels were measured as previously described [20]. A total of 900 μL of doubly distilled water were added to 300 μL of each hydrogel (1.0 wt %) previously prepared into an Eppendorf. The resulting samples were incubated overnight at 25 °C. The swelling ratio, *q*, expressed as percentage, was estimated using the Equation (1), in which *Ws* and *Wd* are the weight of the fully swollen hydrogel obtained after the removal of excess water and of the freeze-dried hydrogels, respectively.

(1) $$q=\frac{\left(Ws-Wd\right)}{Wd}\%$$

### 4.6. Circular Dichroism (CD) Studies

Far-UV CD spectra of all the peptide-based materials were recorded in the 320–190 nm spectral region. Each sample was allocated in a 0.2 mm quartz cell equilibrated at 25 °C. Measurements were performed on a Jasco J-1500-150 spectropolarimeter equipped with a Jasco MCB-100 Mini Water Circulation Bath thermal controller unit (Peltier device), as previously described [29]. 

### 4.7. Fourier Transform Infrared (FT-IR) Spectroscopy

FT-IR spectra of all the 1.0 wt % peptide-based hydrogels were performed on a Jasco FT/IR 4100 spectrometer (Easton, MD, USA) as previously described [20]. After collection in transmission mode, amide I deconvolutions (in the 1600–1700 cm^–1^ region) were automatically returned as emissions by the instrument’s integrated software, namelysecondary structure analysis (SSE) software for Infrared Interpretation and modeling of proteins, version 2.15B.

### 4.8. Thioflavin T (ThT) Spectroscopic Assay

The aggregation behavior of mixed hydrogels was assessed using the ThT assay. Thioflavin T associates rapidly with *β*-aggregated peptides, showing an enhanced emission at 482 nm [54]. Hydrogels (400 μL) were prepared according to the solvent-switch method as reported above. After gels formation, 50 μL of the samples were deposited on a clean coverslip glass, dried, and stained with a 50 μmol·L^−1^ThT solution. The excess solution of ThT on the samples was removed using filter paper, to avoid any corruption of the dried xerogels. The dried stained films were observed under bright-field illumination and in the spectral region of the GFP (Green Fluorescent Protein, *λ*_exc_ = 488 nm, *λ*_em_= 507 nm). Scale bars were acquired with a magnification of 100 μm for all the pictures. Immunofluorescence images were taken with a Leica MICA microhub fluorescence microscope equipped with a 10X objective. 

### 4.9. Birefringence Congo Red (CR) Assay

A dried film of each peptide was prepared by drop-casting ~40 μL of preformed 1.0 wt % peptide hydrogel on a microscope glass slide. Peptide HGs (400 µL) were freshly prepared as already described, by adding 360 μL of a solution containing 2 µL of a saturated CR solution prepared in 20% (*v*/*v*) ethanol saturated with NaCl. Then, the hydrogels were drop-casted and dried overnight on a microscope glass slide at room temperature. The resulting films were observed under bright-field illumination and between crossed polars using an Optech BM80 Pol microscope (Exacta Optech, Modena, Italy). 

### 4.10. Rheological Studies

Rheological measurements of freshly preformed 1.0 wt % hybrid hydrogels (500 μL) were performed with a rotational controlled-stress rheometer (Malvern Kinexus, UK) using a 1.5 cm diameter flat-plate geometry (PU20-PL61). All the analyses were conducted according to the setup previously described [29]. The rheological profiles, reported in Pascal (Pa), were plotted as storage or elastic modulus (G′) and shear loss or viscous modulus (G″).

### 4.11. Scanning Electron Microscopy (SEM)

Morphological analysis of xerogels was carried out by field-emission SEM Phenom_XL (Alfatest, Milan, Italy) as previously described [29]. The analyses were carried out on samples prepared by drop-casting 10 μL of each peptide hydrogel on an aluminum pin stub. 

### 4.12. Cell Lines

Aneuploid immortal keratinocyte cell line HaCat (human) and mouse pre-adipocyte cell line 3T3-L1 were obtained from IRCCS Synlab SDN Biobank (10.5334/ojb.26) and grown in Dulbecco’s modified Eagle medium supplemented with 10% fetal bovine serum and 1% L-glutamine. Cells were incubated at 37 °C and 5% CO_2_ and seeded in 100 mm culture dishes.

### 4.13. Cell Viability and Survival Test

For the adhesion test, both HaCat and 3T3-L1 cell lines were seeded in 96-well plates at a density of 1.5 × 10^3^ cells per well. Before seeding, each well was filled with 50 μL of the indicated hydrogels. In order to test the toxicity of hydrogels’ conditioned media, MTS (3-(4,5-dimethylthiazol-2-yl)-5-(3-carboxymethoxyphenyl)-2-(4-sulfophenyl)-2H-tetrazolium) assay (CellTiter 96 Aqueous One Solution Cell Proliferation Assay, Promega, Italy) was performed. For the cytotoxicity assay, 1.5 × 10^3^HaCat and 3T3-L1 cells per well were seeded in 24-well plates. Hydrogels formed in a hollow plastic chamber (200 μL) were used to obtain the conditioned media [20], [28]. It was observed that the incubation of the media on cells does not cause a change in the color of the media, thus indicating that the pH value (7.5–7.8) is suitable for culturing to be added to the wells. The cells were left to grow under these conditions for three different times (24, 48, and 72 h). At each time point, the cell viability was estimated using the MTS test according to the protocol [55]. The samples were analyzed using the Victor Nivo Multimode Microplate Reader (PerkinElmer) at 490 nm absorbance. The percentage of viable cells in the presence of hydrogels was calculated in comparison to the control cells grown in absence of gel. MTS assays were conducted in triplicate and repeated twice with similar results.

## Figures and Tables

**Table 1:** Formula, theoretical and experimentally found molecular weight (MW), and retention time of the investigated peptides.

Peptide	Formula	MWcalc. (a.m.u.)	MWdeter. (a.m.u.)	t_R_ (min)
Fmoc-FFC	C_36_H_36_N_4_O_5_S	636.7	636.3	21.5
Fmoc-FFS	C_36_H_36_N_4_O_6_	620.6	621.4	19.8
Fmoc-FFT	C_37_H_38_N_4_O_6_	634.7	635.4	20.3

**Table 2:** Characterization of mixed hydrogels at different *w*/*w* ratios. Swelling percentage, gelation time, storage modulus (G′), loss modulus (G″), and Tan*δ*.

System	Ratio (*w*/*w*)	Swelling (%)	G′ (kPa)	G″ (kPa)	Tan*δ*	Gelation Time (min)
Fmoc-FFC/Fmoc-FF	1/5	38.6	13.6	1.0	13.6	75
	1/10	36.2	18.7	1.7	10.9	4.0
	1/20	33.2	16.6	1.6	10	2.6
Fmoc-FFS/Fmoc-FF	1/5	34.0	9.7	1.2	7.8	90
	1/10	34.8	17.3	1.9	9.3	20
	1/20	36.0	13.4	1.7	7.8	6.5
Fmoc-FFT/Fmoc-FF	1/5	37.0	21.5	1.2	17.8	72
	1/10	37.9	21.5	1.6	13.6	48
	1/20	41.0	13.8	1.2	11.6	20

## Data Availability

All data and materials are available on request from the corresponding author. The data are not publicly available due to ongoing research using a part of the data.

## Supplementary Materials

The following supporting information can be downloaded at: https://www.mdpi.com/article/10.3390/gels9110903/s1. Figure S1: Fmoc-FFC characterization (HPLC and ESI). Figure S2: HNMR of Fmoc-FFC. Figure S3: Fmoc-FFS characterization (HPLC and ESI). Figure S4: HNMR of Fmoc-FFS. Figure S5: Fmoc-FFCT characterization (HPLC and ESI). Figure S6: HNMR of Fmoc-FFC. Figure S7: UV-vis profiles of mixed hydrogels (at the different molar ratio) as function of the time. Figure S8: Histogram of swelling test. Figure S9: Time sweeps for mixed hydrogels. Figure S10: Cell viability assays for HaCat and 3T3-L1 line cells.
