# Supplementary material for: Multicomponent Peptide-Based Hydrogels Containing Chemical Functional Groups as Innovative Platforms for Biotechnological Applications
Source: Gels. 2023 Nov 15;9(11):903. doi: 10.3390/gels9110903 (PMC10671135; doi:10.3390/gels9110903)
Supplement: Supplementary file 1 [file gels-09-00903-s001.zip › gels-2698755-supplementary.pdf]

Supplementary Materials

Multicomponent peptide-based hydrogels containing chemical functional groups as innovative platforms for biotechnological applications.

Sabrina Giordano,[1] Enrico Gallo,[2] Carlo Diaferia,[1] Elisabetta Rosa,[1] Barbara Carrese,[2] Nicola Borbone,[1] Pasqualina Liana Scognamiglio,[3] Monica Franzese,[2] Giorgia Oliviero,[4] Antonella Accardo[1]

[1]Department of Pharmacy, University of Naples "Federico II", Via D. Montesano, 49- 80131-Naples, Italy.

[2]IRCCS Synlab SDN, Via Gianturco 113, Naples, 80143, Italy

[3]Department of Sciences, University of Basilicata, Via dell'Ateneo Lucano 10, 85100-Potenza, Italy

[4]Department of Molecular Medicine and Medical Biotechnologies, University of Naples Federico II, Via S. Pansini 5, 80131 Naples, Italy

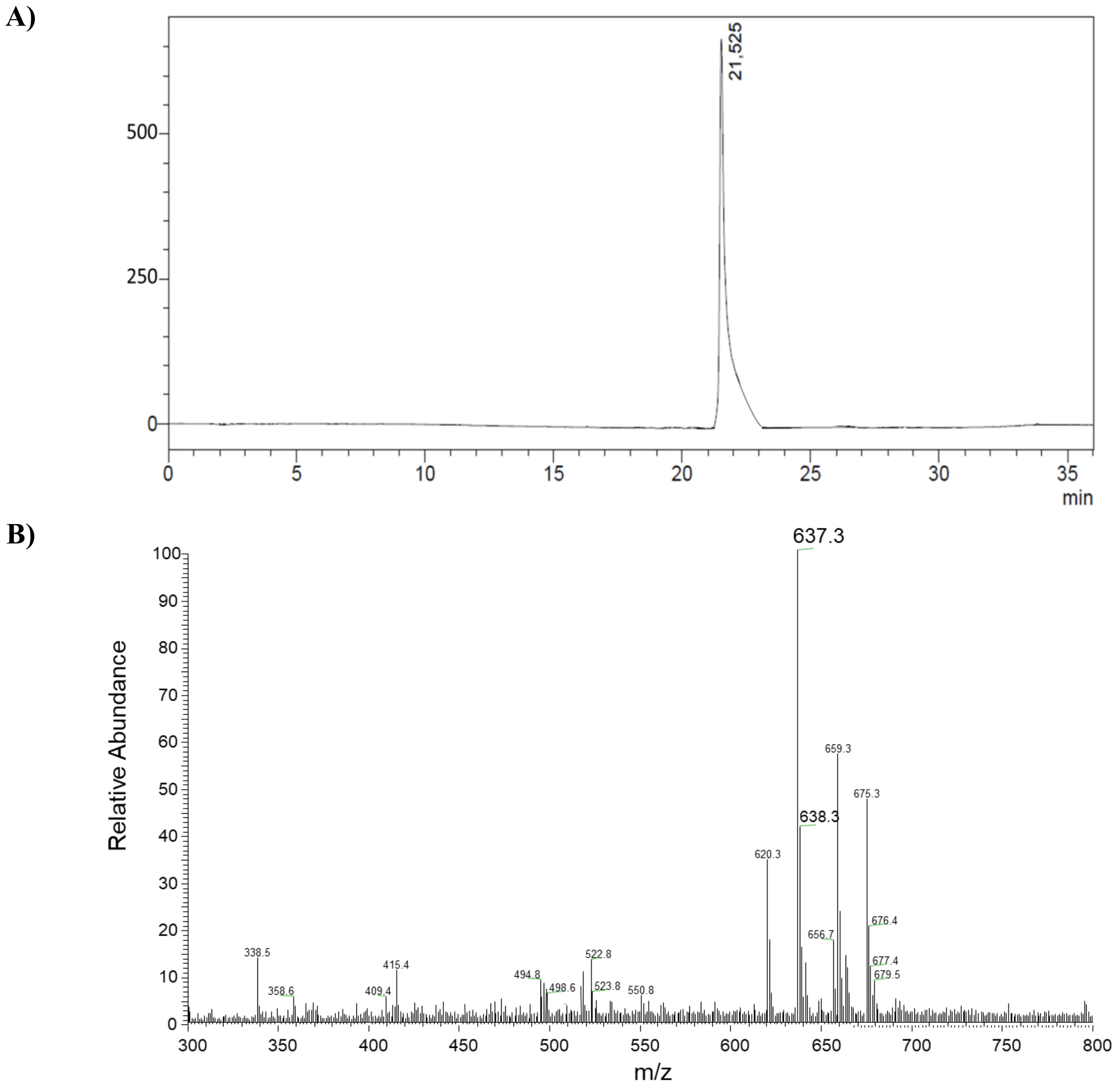

Figure S1: Physicochemical characterization of Fmoc-FFC peptide: A) RP-HPLC chromatography and B) ESI mass spectrum

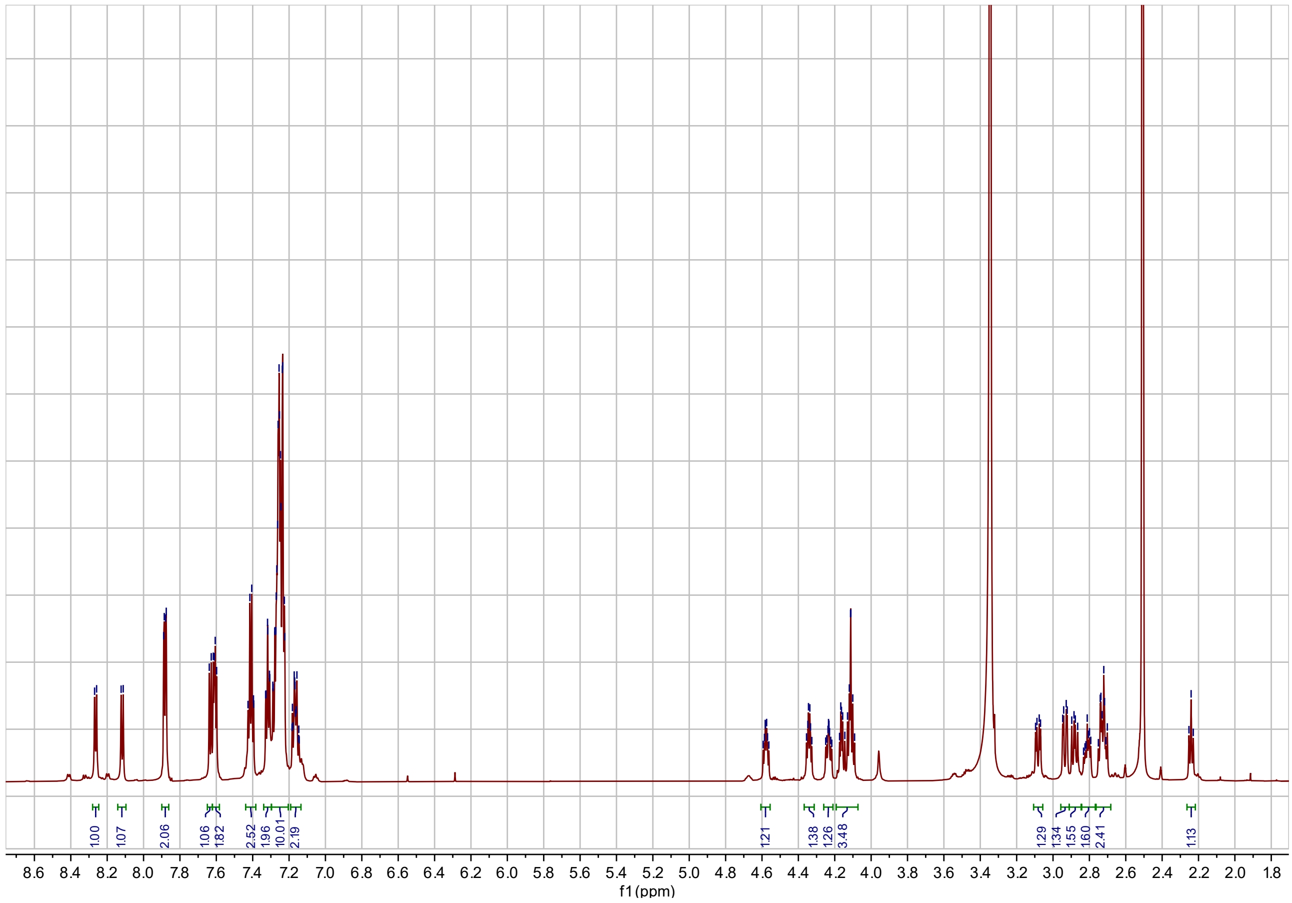

Figure S2: HNMR spectrum of Fmoc-FFC

A)

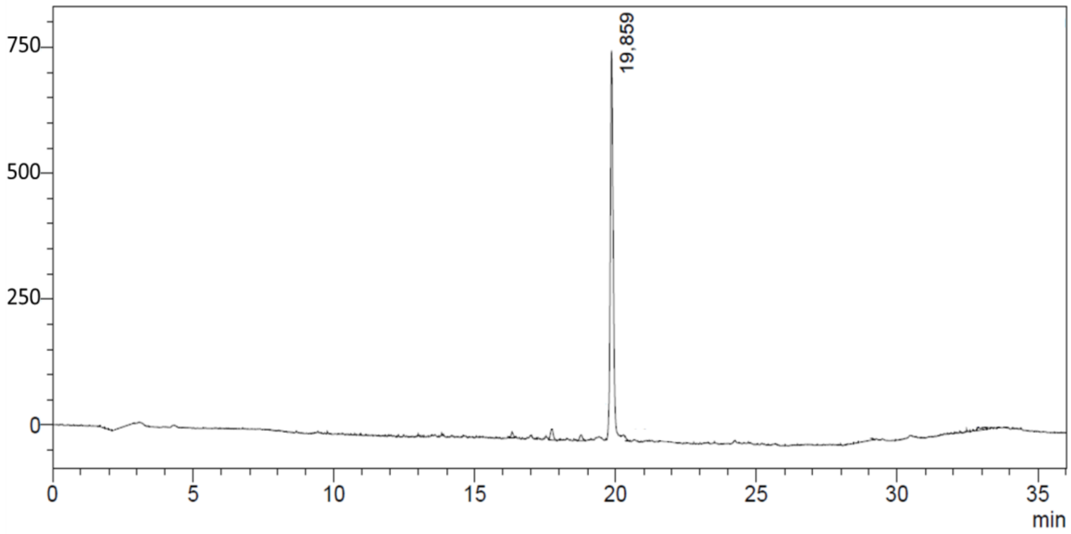

B)

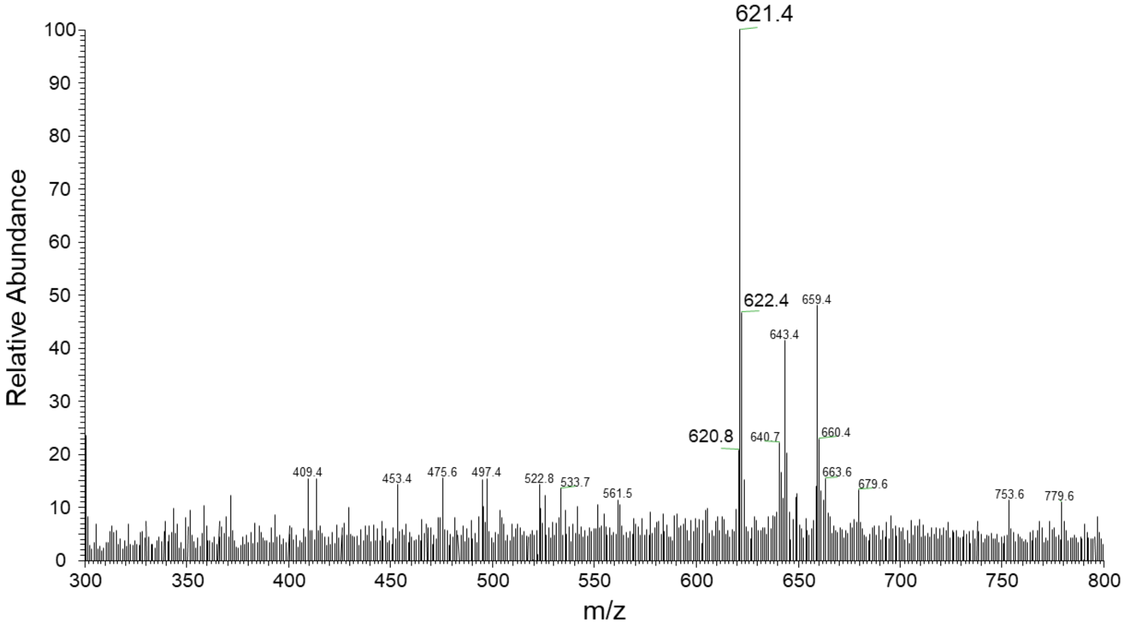

Figure S3: Physicochemical characterization of Fmoc-FFS peptide: A) RP-HPLC chromatography and B) ESI mass spectrum

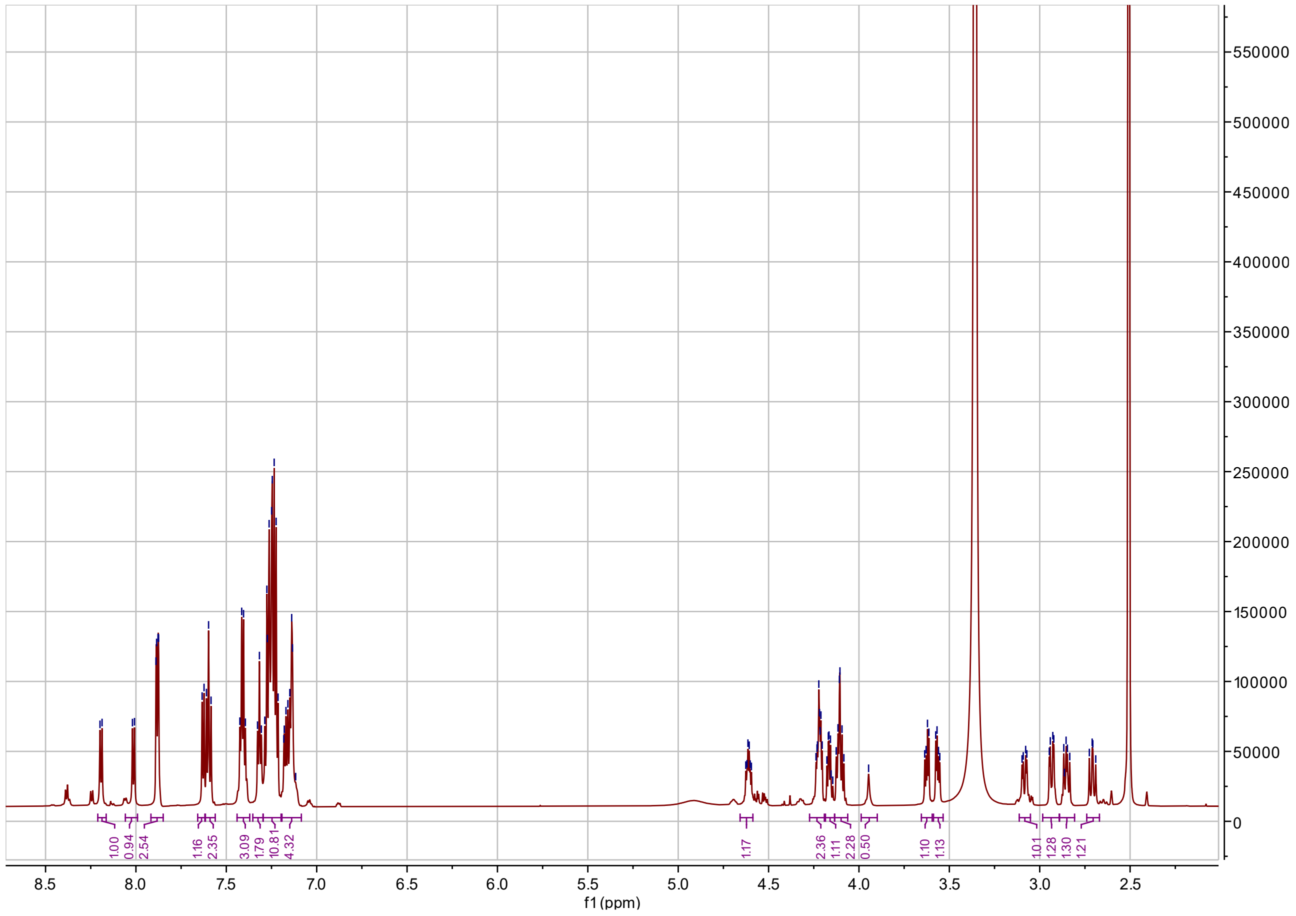

Figure S4: HNMR spectrum of Fmoc-FFS

A)

B)

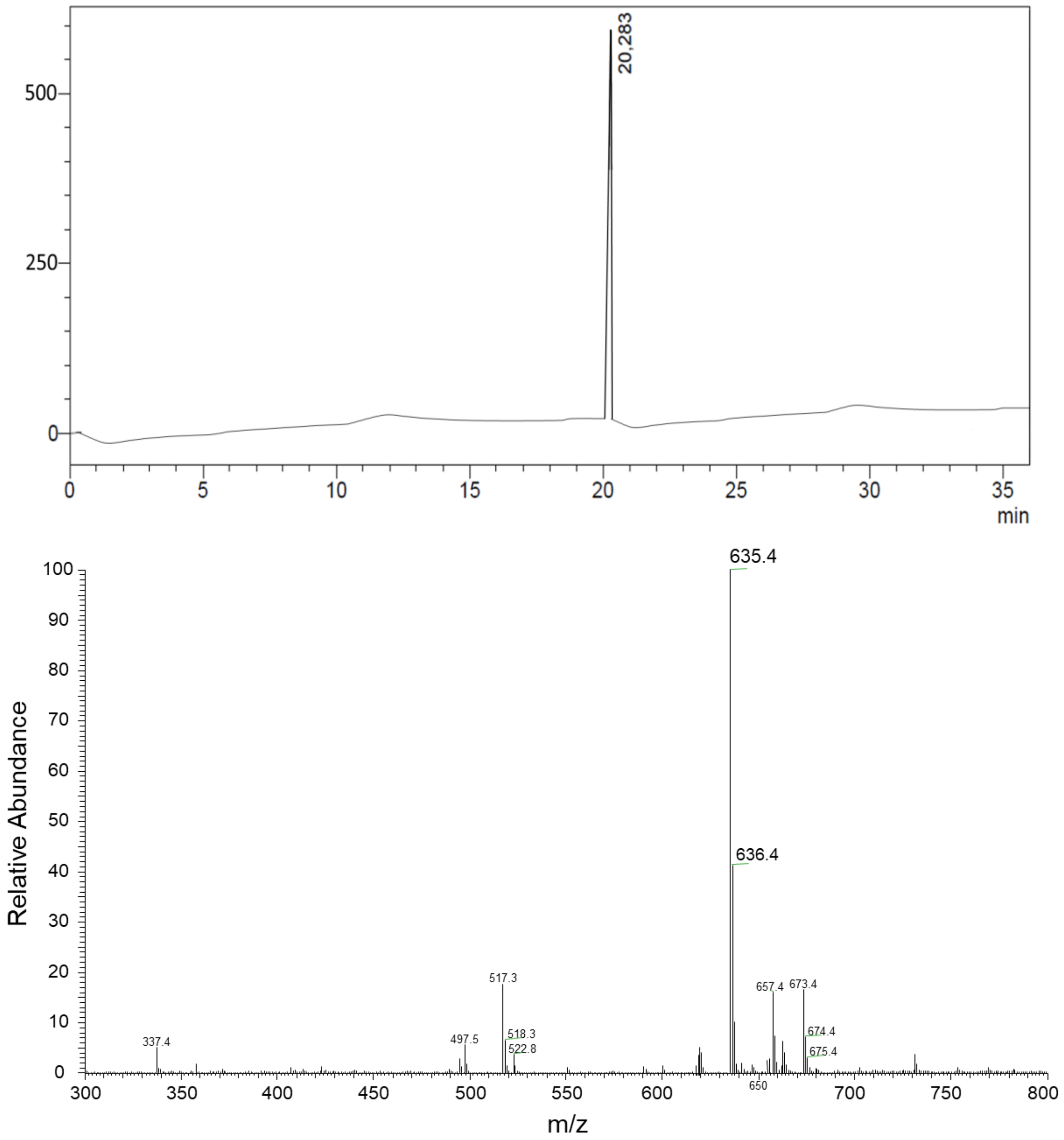

Figure S5: Physicochemical characterization of Fmoc-FFT peptide: A) RP-HPLC chromatography and B) ESI mass spectrum

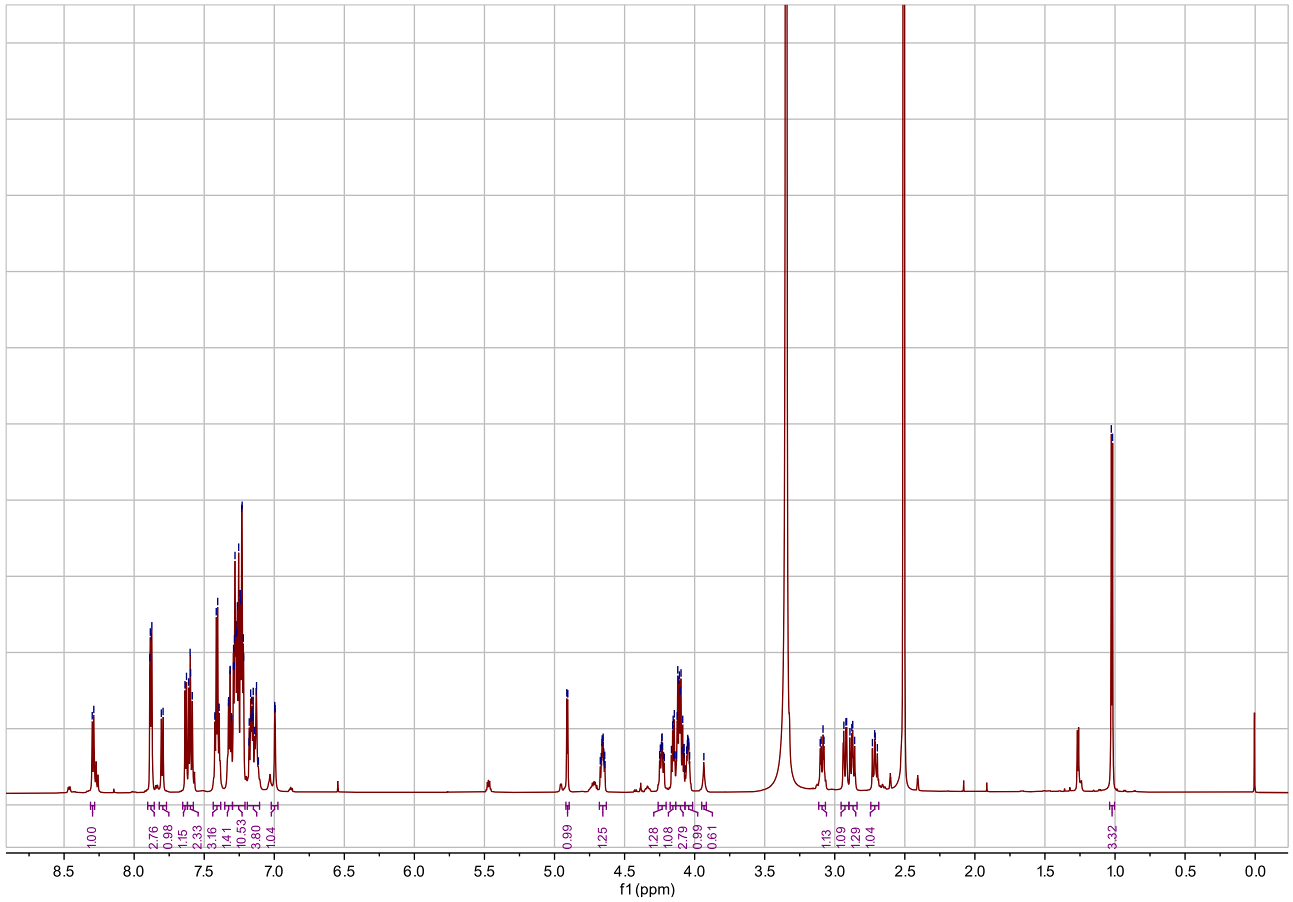

Figure S6: HNMR spectrum of Fmoc-FFT

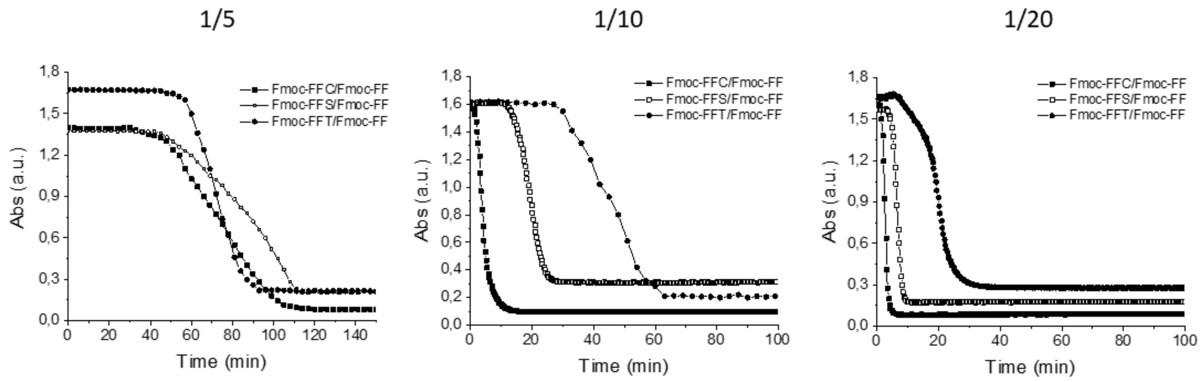

Figure S7: UV-vis profiles of mixed hydrogels (at the different molar ratio) in the long range as function of the time.

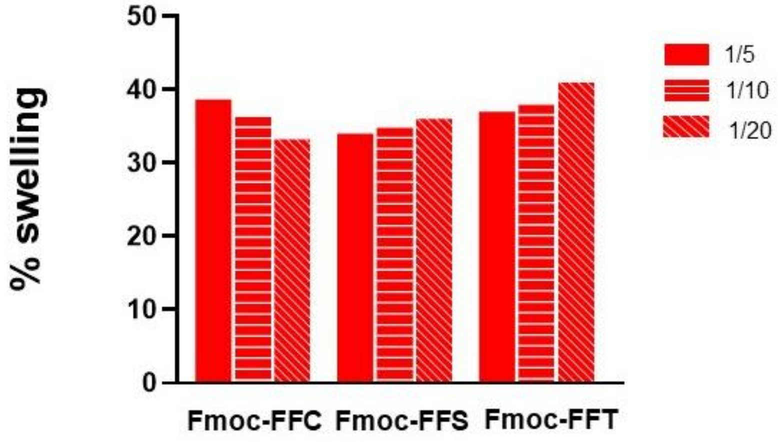

Figure S8: Swelling percentage for mixed hydrogels containing Fmoc-FFC, Fmoc-FFS or Fmoc-FFT at 1/5, 1/10 and 1/20 w/w ratio.

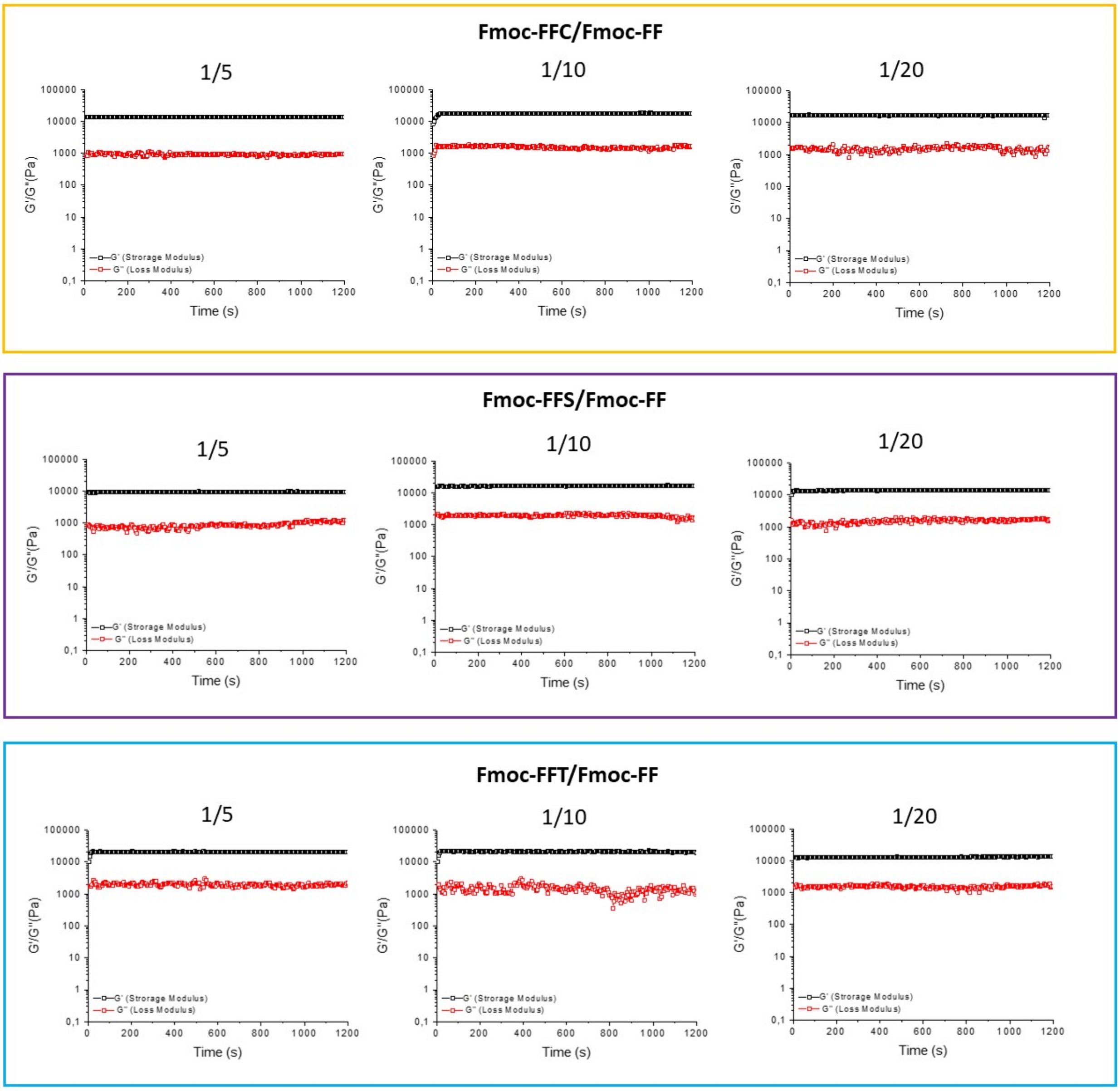

Figure S9: Time sweep (20 minutes) for mixed hydrogels Fmoc-FFC/Fmoc-FF, Fmoc-FFS/Fmoc-FF or Fmoc-FFT/Fmoc-FF at 1/5, 1/10 and 1/20 w/w ratio. Rheological analysis is reported in terms of G' (Storage modulus) and G'' (Loss modulus).

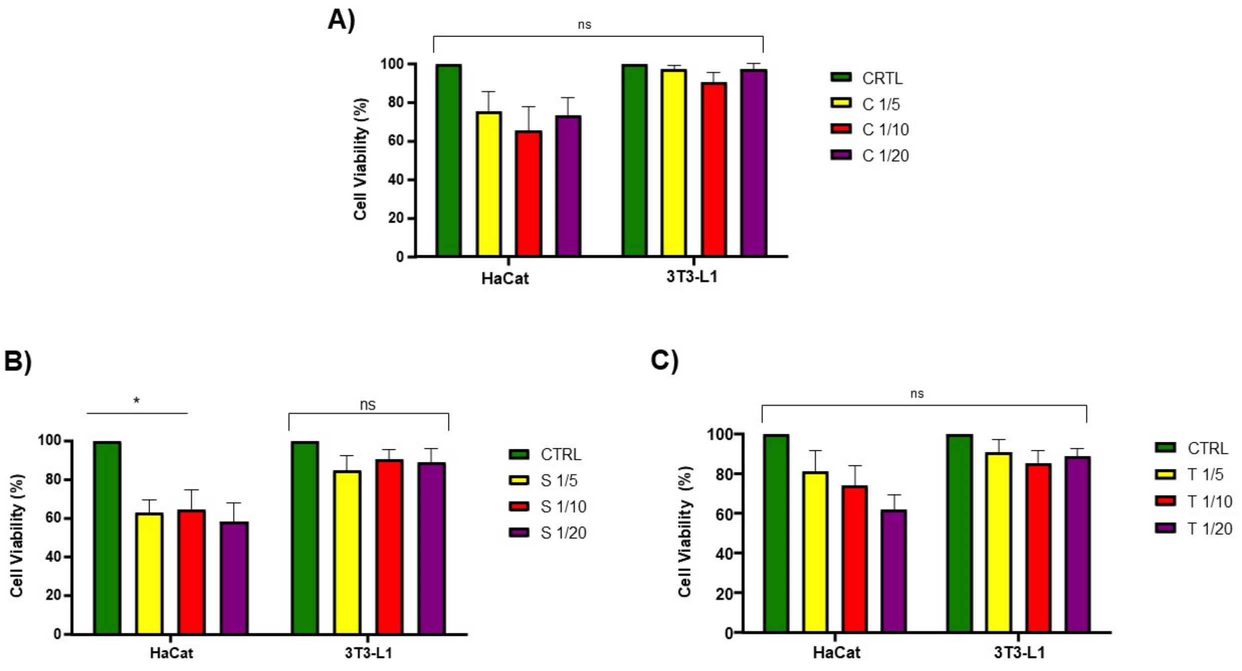

Figure S10: Cell viability assay. MTS assay of HaCat and 3T3-L1 cells treated for 24 h with hydrogels conditioned medium (Fmoc-FFC/Fmoc-FF (A) Fmoc-FFS/Fmoc-FF (B) and Fmoc-FFT/Fmoc-FF (C)). * $p < 0.05$ *versus* control cells
